# Reply

**Published:** 2010

**Authors:** Attia Z. Taha

**Affiliations:** Family and Community Medicine Department, College of Medicine, University of Dammam, Dammam, Saudi Arabia aztaha@hotmail.com

We would like to thank Dr. Sreedharan for his comments on our article.

Concerning the prevalence of shisha smoking, there is no contradiction regarding the results. We have shown the detailed smoking habits of students in Table 1. This makes it clearer than many other studies which show only cigarette or shisha smoking without considering that some people smoke both cigarettes and shisha. In this study we are only concerned with shisha smoking. Therefore, the prevalence of shisha will include both shisha only (n=32) and both cigarettes and shisha (n=15) which will amount to 47 out of 371 and this gives a prevalence of 12.6%. By the first sentence of the Discussion we mean “the overall prevalence of shisha and shisha plus cigarette smoking”. Table 1 will remove the confusion, which some readers might have when reading the first sentence in the Discussion.

With regard to the comment about nonrespondents in Table 3, we performed the Chi-square test with “Refused to answer” included (Chi-square=14.1; 5df; *P*=.015) and with “Refused to answer” excluded (Chi-square=14.1; 4 df; *P*=.007) and the result was the same. Thus, we decided to include them as those were important variables that did not affect the result and we did not consider them as nonrespondents to make it more clear to the reader. In a brief report you do not need to explain all details concerning every step of data analysis in the text.

With regard to the comment about calculation of the denominator among mothers with education, the use of column total or row total depends on the author’s objective from displaying the set of data. You are right to use the row total when calculating the prevalence of shisha use among mothers with education. However, in Table 3 we are comparing the levels of the mother’s education (%) within shisha smoking status. This will give a clear picture of the relation between smoking status and educational level. Using either column total or row total will not change the result of association.

The same principle applies to the type of college in Table 3. It is obvious from Table 3 to reach your conclusion (that a high prevalence of shisha use is seen among dental students and least among the medical students) when calculating the prevalence of shisha smoking among students in each college. Our aim was to highlight the proportion of shisha smokers in relation to the total number of students who smoke.

